# Genome-wide analysis of eukaryote thaumatin-like proteins (TLPs) with an emphasis on poplar

**DOI:** 10.1186/1471-2229-11-33

**Published:** 2011-02-15

**Authors:** Benjamin Petre, Ian Major, Nicolas Rouhier, Sébastien Duplessis

**Affiliations:** 1 INRA†/Nancy Université, Unité Mixte de Recherche 1136 'Interactions Arbres/Micro-organismes', Centre INRA de Nancy, F-54280 Champenoux, France; 2 Plant Research Laboratory, 122 Plant Biology Laboratory, Michigan State University, East Lansing, Michigan, 48864, USA

## Abstract

**Background:**

Plant inducible immunity includes the accumulation of a set of defense proteins during infection called pathogenesis-related (PR) proteins, which are grouped into families termed PR-1 to PR-17. The PR-5 family is composed of thaumatin-like proteins (TLPs), which are responsive to biotic and abiotic stress and are widely studied in plants. TLPs were also recently discovered in fungi and animals. In the poplar genome, TLPs are over-represented compared with annual species and their transcripts strongly accumulate during stress conditions.

**Results:**

Our analysis of the poplar TLP family suggests that the expansion of this gene family was followed by diversification, as differences in expression patterns and predicted properties correlate with phylogeny. In particular, we identified a clade of poplar TLPs that cluster to a single 350 kb locus of chromosome I and that are up-regulated by poplar leaf rust infection. A wider phylogenetic analysis of eukaryote TLPs - including plant, animal and fungi sequences - shows that *TLP *gene content and diversity increased markedly during land plant evolution. Mapping the reported functions of characterized TLPs to the eukaryote phylogenetic tree showed that antifungal or glycan-lytic properties are widespread across eukaryote phylogeny, suggesting that these properties are shared by most TLPs and are likely associated with the presence of a conserved acidic cleft in their 3D structure. Also, we established an exhaustive catalog of TLPs with atypical architectures such as small-TLPs, TLP-kinases and small-TLP-kinases, which have potentially developed alternative functions (such as putative receptor kinases for pathogen sensing and signaling).

**Conclusion:**

Our study, based on the most recent plant genome sequences, provides evidence for *TLP *gene family diversification during land plant evolution. We have shown that the diverse functions described for TLPs are not restricted to specific clades but seem to be universal among eukaryotes, with some exceptions likely attributable to atypical protein structures. In the perennial plant model *Populus*, we unravelled the TLPs likely involved in leaf rust resistance, which will provide the foundation for further functional investigations.

## Background

Plants respond to challenge from pathogens by activating an inducible protein-based defense system that includes 17 families of pathogenesis-related (PR) proteins termed PR-1 to PR-17 [1,2]. Proteins of the PR-5 family have high sequence identity with thaumatins, which are sweet-tasting proteins isolated from the West African shrub *Thaumatococcus daniellii *and are thus referred to as thaumatin-like proteins (TLPs) [3]. For decades, TLPs have been studied extensively in plants for their antifungal properties. The recent identification of TLPs in animals [4] and fungi [5] indicates that these proteins are more widely distributed and not only restricted to plants [6].

Molecular studies of TLP expression, localisation and activity support a role for TLPs in host defense during pathogen infection. TLP up-regulation has been described in many higher plants infected by pathogens such as bacteria, oomycetes and fungi [7,8]. Localisation studies revealed that plant pathogen-inducible TLPs are secreted into the apoplast [9,10]. More than 20 TLPs from animals, fungi and plants have been shown to exhibit an antifungal activity [7], although the mechanisms by which TLPs exert this activity remain unclear. Several antifungal modes of action have been described such as membrane permeabilization [11], β-glucan binding and degradation [5], inhibition of enzymes such as xylanases [12], α-amylase, or trypsin [13], as well as an apoptosis-inducing mechanism reported in yeast [14]. Other functional properties have been reported for TLPs, including antifreeze activity [15], protection from abiotic stress [16] and binding to proteins such as actin, viral CMV-1 protein, yeast glycoproteins and G-Protein Coupled Receptor (GPCR) or to hormones such as cytokinins [7].

Most typical TLPs described to date have a molecular weight ranging from 20 to 26 kDa, and generally possess 16 conserved cysteine residues that form eight disulfide bonds [17]. Recently, small TLPs (sTLPs) have been identified in monocots and conifers. These are characterized by a smaller molecular weight (around 17 kDa) and only 10 conserved cysteine residues that form five disulfide bonds [18-20]. Seven TLP structures have been solved so far, revealing a strongly conserved 3D organisation with a characteristic acidic cleft domain that comprises the five highly conserved amino acids REDDD that are dispersed in the primary sequence [21]. Despite good conservation of these amino acids in sTLP primary sequences, they do not organize into an acidic cleft at the 3D level [22]. Unusual TLP and protein kinase fusion proteins referred to as PR5-kinase or TLP-kinase (TLP-K) have also been reported in a few plant species [23,7].

The analysis of the *Populus trichocarpa *'Nisqually-1' genome revealed a substantial over-representation of genes encoding disease resistance proteins compared with annual species such as *Arabidopsis thaliana*, and this increase is not solely attributable to the genome expansion in *Populus *[24]. In particular, 55 putative *TLP *genes were initially identified in *P. trichocarpa *versus 24 for *A. thaliana *[24]. *Populus *spp. are economically important and hybrid poplars in particular are used extensively worldwide for wood production. Breeding programs particularly target resistance to *Melampsora *spp. fungi, which are responsible for leaf rust, a major disease of poplars that severely impacts tree growth and wood production [25]. With the availability of both *P. trichocarpa *and *M. larici-populina *genome sequences, the biotrophic poplar-rust interaction is emerging as a model pathosystem in forest biology [26]. Several transcriptome-based studies revealed transcriptional reprogramming in poplar leaves infected by *Melampsora *spp., including the up-regulation of many PR proteins [26]. In particular, transcript profiling of poplar leaves during an incompatible interaction (i.e. host-specific resistance) with *M. larici-populina *established a set of host-defense marker genes, including several TLPs [27].

The present study describes the annotation of 42 *TLP *gene models in the *P. trichocarpa *'Nisqually-1' genome version 2.0. In addition, comparison of expression studies conducted on poplar subjected to biotic (i.e. *Melampsora *spp. infection) and abiotic stresses identified stress-responsive clades. The comparison of 598 complete eukaryote TLP amino acid sequences, of which 410 come from the 18 plant genome sequences currently available, allowed us to establish a link between function and phylogeny by systematically mapping functional data mined from the literature to the phylogenetic tree. *In silico *structural analysis confirmed that, with the exception of sTLPs, the acidic cleft domain is strongly conserved among eukaryote TLPs.

## Results

### Annotation, phylogeny, genomic distribution and gene expression of poplar TLPs

In contrast to Tuskan and collaborators [24], we identified a total of 59 putative *TLP *genes in the *P. trichocarpa *'Nisqually-1' genome version 1.1. In version 2.0 of the genome, now integrated in the Phytozome portal [28,29], 17 of these *TLP *gene models are not validated. These 17 invalidated models include 11 predicted alleles that were previously considered to be independent genes and six probable pseudogenes that are interrupted by stop codons (Additional file 1). The remaining 42 *TLP *genes that are validated in version 2.0 of the genome comprise 38 typical TLPs and four genes with strong homology to TLP-K from *A. thaliana*, including fusion to a putative protein kinase (Pfam: PF00069) ([23], Additional file 2).

**Figure 1:**
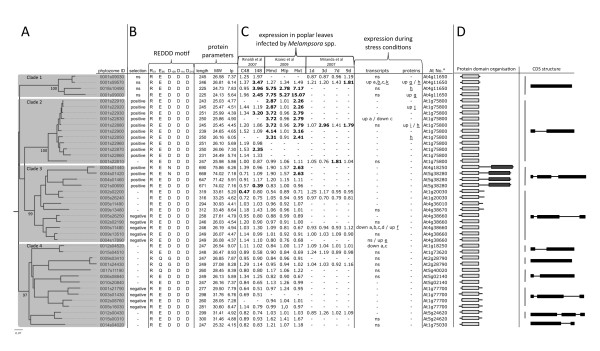
**Thaumatin-like proteins (TLPs) from poplar**. (A), Neighbour-joining tree of *Populus trichocarpa *TLPs. Branch lengths are proportional to phylogenetic distances. (B), Protein characteristics and natural selection of poplar TLPs. MW: mass weight in kDa; Ip: predicted iso-electric point; ns: neutral selection [39]. (C), Regulation of poplar TLPs during stress. Transcriptome analyses of 3 different studies on poplar leaves infected by *Melampsora *spp. are summarized [27,30,31]. Changes considered to be significant by the respective authors are in bold. I48: incompatible interaction at 48 hour post-inoculation (hpi); C48: compatible interaction at 48 hpi; Mmd: compatible interaction at 6 dpi; Mlp: compatible interaction at 6 dpi; Mxt: Mmd+Mlp; 1d, 3d, 7d, 9d: compatible interaction respectively at 1, 3, 7 and 9 dpi. Summarized data for expression during stress conditions were mined from the PopGenIE database [58] (non-underlined letters) or from the literature (underlined letters). 'up': up-regulated gene or increased protein accumulation; 'down': down-regulated gene; ns: no significant regulation; a letter alone indicates that the corresponding protein has been reported but no regulation information is available; a to d: ozone, UV, drought and cold stress respectively; e: wind exposed leaves; f: wounding; g: *Populus*/*Melampsora *compatible interaction; h: sap extract; i: sap extract after wounding; j: wood regeneration; k: copper stress. Corresponding references: [60,65-71] (D), Protein domain organisation and CDS structure. Light grey box: thaumatin domain; dark grey box: protein kinase domain; black box: exon. '-' in (A), (B) and (C) indicates missing information. ^a^Accession number of the best *Arabidopsis thaliana *homolog.

A phylogenetic tree constructed with the validated poplar TLPs reveals four well-defined clades, numbered here from 1 to 4. Among these clades, the REDDD residues are highly conserved with only small variations for five TLPs (Figure 1). The size of the proteins varies from 225 to 319 amino acids (~24 to 34 kDa) for the 38 typical TLPs and is approximately 650 amino acids (~73 kDa) for the four TLP-Ks. The predicted isoelectric points vary from 4.15 to 9.07 and correspond well with the TLP phylogeny (Figure 1). Analysis of the protein domain organisation showed that the thaumatin domain (Pfam: PF00314) covers almost 95% of the entire mature TLPs, except 10 TLPs in clades 3 and 4 that have approximately 40 additional amino acids in their C-terminal region. The four TLP-Ks are grouped in a specific branch of clade 3, suggesting that they are monophyletic in poplar. The gene structure of poplar *TLPs *is well conserved within clades 1-3, with genes belonging to clade 1 formed by a single exon, *TLPs *from clade 2 by two exons and *TLPs *of clade 3 by three exons (Figure 1); clade 4 is an exception with genes composed of one, two or three exons.

**Figure 2:**
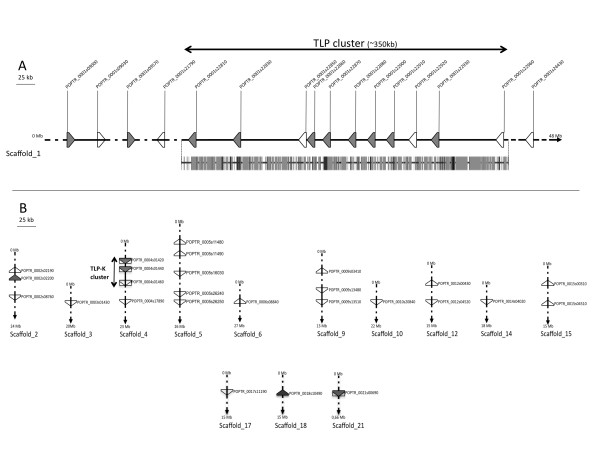
**Representation of genomic loci of *TLP *genes in the genome of *Populus trichocarpa *'Nisqually-1'**. (A), Position of *TLP *genes on scaffold 1. Transposable element coverage of the TLP cluster is presented below scaffold 1 (dark grey: LTR-retrotransposon; light grey: DNA transposon). (B), position of *TLP *genes on scaffolds 2 to 21. Black lines: scaffolds; triangles: *TLP *genes; triangles in rectangles: *TLP-kinase *genes. Grey and white triangles respectively correspond to regulated and non-regulated genes in rust-infected poplar leaves as shown in Figure 1.

The version 2.0 of the *P. trichocarpa *genome incorporates a greatly improved physical map compared with version 1.1. This helped localise 41 of the 42 annotated *TLP *genes on 13 of the 19 chromosomes (i.e. scaffolds 1 to 19 on the Phytozome portal [29]) (Figure 2). Scaffold 1 contains 16 *TLP *genes, including all 11 *TLP *genes from clade 2 which are located within a 350 kb segment that encodes *TLPs *exclusively. We named this region the TLP cluster. Transposable elements (TE) cover 52% of this 350 kb region, with a particular over-representation of long terminal repeat (LTR) Gypsy elements that cover 37% of the cluster (Figure 2 and Additional file 3).

Results compiled from three different previously published transcriptome analyses of poplar leaves infected by *Melampsora *spp. fungi [27,30,31] indicate that, of the 42 *TLP *genes, 14 are significantly up-regulated and two are significantly down-regulated (Figure 1). Among the 14 up-regulated TLP transcripts, 12 belong to clades 1 and 2 and 11 of these are located on scaffold 1 (Figure 1 and 2). Interestingly, five *TLP *genes are up-regulated during an incompatible poplar/rust interaction, of which three are grouped in clade 1. Under abiotic stress conditions, five poplar TLP transcripts showed differential accumulation. In addition, six TLPs were identified by different proteomic studies, of which four were shown to accumulate during biotic or abiotic stress (Figure 1). More specifically, the *PopTLP1 *gene (*P. trichocarpa *geneID Poptr_0001s09570) from clade 1 is associated with several biotic and abiotic stresses and we confirmed with a detailed time-course analysis by RT-qPCR that *PopTLP1 *expression increases in poplar leaves challenged by *M. larici-populina *(Additional file 4).

### TLPs in green plant genome sequences

We performed an exhaustive genomic analysis of plant *TLPs *by collecting *TLP *gene models from 18 sequenced plants available at the Phytozome portal [29]. Models encoding proteins with an incomplete thaumatin domain were ignored (Table 1). A single but incomplete *TLP *gene was identified in the unicellular green algae *Chlamydomonas reinhardtii*, which represents the evolutionary starting point of viridiplantae, and thus makes the origin of complete TLPs in the green lineage unclear (Table 1). Three complete *TLP *genes were identified in the moss *Physcomitrella patens *and 12 were found in the vascular plant *Selaginella moellendorffii*, indicating that an important gene expansion occurred in the transition from bryophytes to tracheophytes. Among the 15 angiosperm genomes, the *TLP *gene number varies from 16 in the barrel clover *Medicago truncatula *to 42 in the black cottonwood *P. trichocarpa*, whereas *A. thaliana *has 22 *TLP *genes. An average of 26 *TLP *genes are present in angiosperms, with similar numbers of *TLPs *in dicots or monocots (Table 1). sTLP-encoding genes were identified exclusively in monocots (from 2 in *Zea mays *to 9 in *Sorghum bicolor*), whereas TLP-Ks have been identified in both monocots and dicots, although dicot TLP-Ks were restricted to the *A. thaliana *and *P. trichocarpa *genomes. To identify the genes that are most similar to TLP-Ks in the remaining dicots, we performed homology searches with the kinase domain of TLP-Ks and retrieved only lectin-kinase genes, confirming the absence of *TLP-Ks *in these dicot genomes (data not shown). In *S. bicolor*, a small-TLP-kinase (here termed sTLP-K) composed of a N-terminal sTLP domain and a C-terminal protein kinase domain, separated by a predicted transmembrane (TM) domain, was identified (Additional file 5). The origin of this arrangement is puzzling and has apparently evolved independently of TLP-Ks. To our knowledge, this is the first report of such a domain organisation.

**Table 1:** *TLP *gene content in sequenced plant species

organism	code	common organism name	phylum	class	order	TLP blast result^a^	complete TLP domain^c^	small-TLP/TLP-K^d^
*Chlamydomonas reinhardtii*	Chlre	Green algae	Chlorophyte	Chlorophyceae	Volvocales	1	0	0/0
*Physcomitrella patens*	Phypa	Moss	Bryophyte	Bryopsides	Funariales	5	3	0/0
*Selaginella moelledorffii*	Selmo	Lycophyte	Tracheophyte	Sellaginellopsides	Selaginellales	18	12	0/0
*Oryza sativa*	Orysa	Rice	Angiosperm	Monocotyledon	Cyperales	37	26	4/1
*Brachypodium distachyon*	Bradi	Purple false brome	Angiosperm	Monocotyledon	Poales	32	24	3/2
*Sorghum bicolor*	Sorbi	Sorghum	Angiosperm	Monocotyledon	Poales	45	36	9/1(1^e^)
*Zea mays*	Zeama	Maize	Angiosperm	Monocotyledon	Poales	38	29	2/2
*Mimulus guttatus*	Mimgu	Common monkey-flower	Angiosperm	Dicotyledon	Lamiales	33	23	0/0
*Vitis vinifera*	Vitvi	Grapevine	Angiosperm	Dicotyledon	Rosales	27	18	0/0
*Carica papaya*	Carpa	Papaya tree	Angiosperm	Dicotyledon	Brassicales	18	16	0/0
*Arabidopsis thaliana*	Arath	Thale cress	Angiosperm	Dicotyledon	Brassicales	30	22	0/3
*Cucumis sativus*	Cucsa	Cucumber	Angiosperm	Dicotyledon	Cucurbitales	29	28	0/0
*Glycine max*	Glyma	Soya	Angiosperm	Dicotyledon	Fabales	58	38	0/0
*Medicago truncatula*	Medtr	Barrel clover	Angiosperm	Dicotyledon	Fabales	21	16	0/0
*Prunus persica*	Prupe	Peach tree	Angiosperm	Dicotyledon	Rosales	37	28	0/0
*Manihot esculenta*	Manes	Manioc	Angiosperm	Dicotyledon	Malpighiales	34	27	0/0
*Ricin communis*	Ricco	Castor oil plant	Angiosperm	Dicotyledon	Malpighiales	24	22	0/0
*Populus trichocarpa*	Poptr	Poplar	Angiosperm	Dicotyledon	Malpighiales	59^b^	42	0/4

^a^Number of putative *TLP *genes identified by amino acid homology searches of plant genome sequences on the Phytozome portal [29].

^b^Number of putative *TLP *genes identified from version 1.1 of the *Populus trichocarpa *'Nisqually-1' genome on the JGI website [55].

^c^TLP sequences with a complete thaumatin domain.

^d^Proportion of sTLP and TLP-K with a complete thaumatin domain.

^e^small-TLP/kinase domain fusion (sTLP-K).

### Eukaryote TLPs: linking phylogeny with protein structure and function

**Figure 3:**
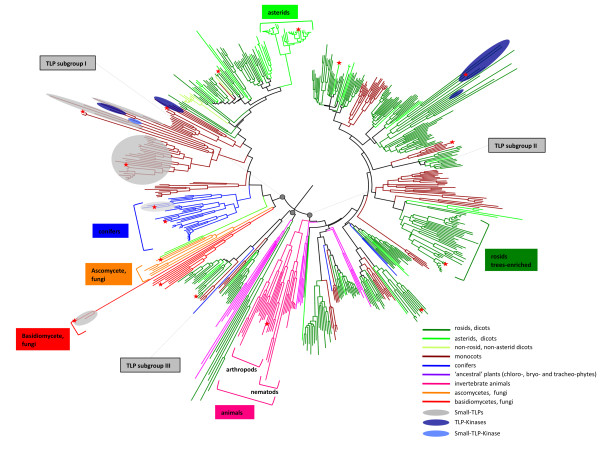
**Neighbour-joining tree of 598 thaumatin domains of TLP sequences from 100 eukaryote species**. Branch lengths are proportional to phylogenetic distances. For clarity, protein names are not indicated but can be retrieved from individual phylogenetic trees of subgroups I, II and III respectively in Figure 5, Additional files 9 and 10. Red stars indicate sequences used for the alignment presented in Figure 4. Annotations of subgroups and clades are discussed in the text.

To achieve an accurate and complete phylogeny of eukaryote TLPs, we retrieved an additional 188 sequences with a complete thaumatin domain from the NCBI protein database [32] and combined them with the 410 plant sequences that we identified earlier (Additional file 6). These include several sequences from fungi (basidiomycetes and ascomycetes) and invertebrate animals (nematods and arthropods), as well as other plants from mainly the asterid and conifer divisions. We report for the first time the identification of *sTLP *genes in basidiomycetes, precisely in the pucciniales *M. larici-populina *and *Puccinia graminis *f.sp. *tritici*. Fungal sTLPs appear to be monophyletic, suggesting that sTLPs evolved independently in pucciniales, monocots and conifers or that sTLPs were lost during evolution from other phyla such as dicots and animals (Additional file 7). Overall, a total of 598 sequences were retrieved from 100 different species (12 animals, 12 fungi and 76 green plants) and were used for comparative genomic analyses. The phylogeny of these eukaryote TLPs reveals three major monophyletic groups (Figure 3). TLP subgroup I consists of 211 sequences and includes highly specific clades, such as a fungal clade containing TLPs from both ascomycetes and basidiomycetes, as well as plant clades that are specific to conifers, monocots, monocot sTLPs, monocot TLP-Ks, dicots or asterids. TLP subgroup II is composed of 341 sequences and includes an animal-specific clade with distinct sub-clades for nematodes and arthropods. Because of their over-representation, a large clade of plant sequences constitutes the vast majority of TLP subgroup II, with several subclades composed of relatively balanced numbers of monocot and dicot sequences (Figure 3). TLP subgroup II notably includes a clade enriched in rosid and tree TLPs that in particular contains the poplar TLP cluster. Dicot TLP-Ks also belong to TLP subgroup II. TLP subgroup III contains only 46 sequences from 20 different plant species, with a large number of sequences from the vascular plant S. *moellendorffii *(Figure 3).

**Figure 4:**
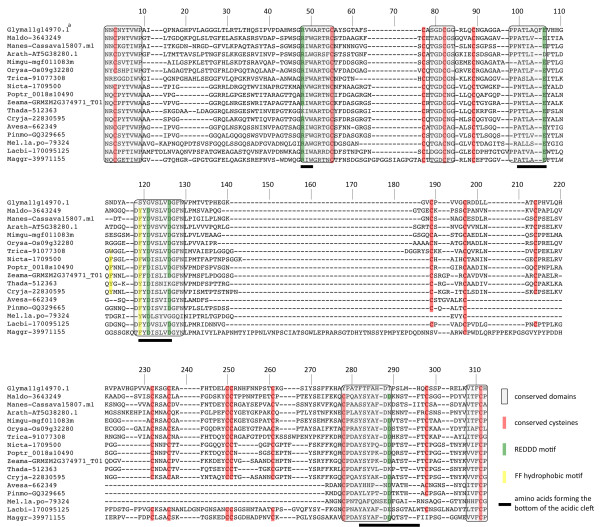
**Alignment of thaumatin domains of selected eukaryote TLPs**. Amino acid sequence comparison was carried out with ClustalW on MEGA 4 software with the parameters described by [6]. The alignment was then adjusted manually when necessary. ^a^Complete protein reference: Glyma-Glyma11g14970.1.

An alignment with 18 representative TLP sequences from the major sub-clades shows the diversity of eukaryote TLPs (Figure 4). The thaumatin domain of ascomycetes is almost 30% longer than that of typical TLPs (~280 versus ~215 amino acids), mainly due to three insertions in less-conserved regions of the domain. By contrast, sTLPs are almost 30% smaller than typical TLPs (~150 versus ~215 amino acids) due to a large deletion. The 16 cysteine residues (10 for sTLPs) are extremely well conserved, except for 1-2 residues in ascomycete and basidiomycete sTLPs and in some animal sequences (Figure 4). The REDDD motif or its equivalent (i.e. amino acids with similar biochemical properties) is fully conserved in 13 of the 18 representative sequences. Similarly, the amino acids forming the bottom of the acidic cleft and those at each extremity of the thaumatin domain are generally well conserved.

**Figure 5:**
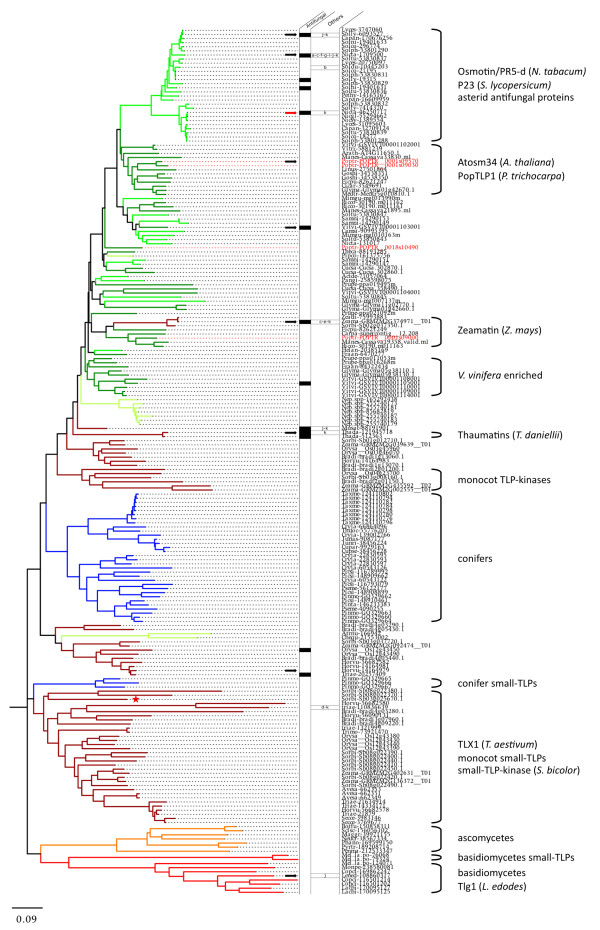
**Neighbour-joining tree of the 211 thaumatin domains of TLP subgroup I**. Functionally characterized TLPs and corresponding functions are indicated. Poplar sequence names are in red. The 5 letter code before each protein ID corresponds to the 3 first letters of the genus name followed by the 2 first letters of the species name. The red arrow indicates PR-5d used for 3D structure alignment and black arrows indicate sequences used for alignment mapping on 3D Structure (see Figure 6). The red star indicates the Small-TLP-Kinase from *Sorghum bicolor *(Sb03g025670). The two columns successively indicate proteins with demonstrated antifungal activity and other functions. a: protection against abiotic stress; b: antifreeze activity; c: membrane permeabilization activity; d: xylanase inhibitor; e: α-amylase/trypsin inhibition; f: apoptosis-inducing in yeast; g: GPCR binding; h: CMV1-a binding; i: glycoprotein binding; j: endo-β-1,3-glucanase activity; k: solved 3D structures. References corresponding to these data are summarized in Additional file 8. Branch lengths are proportional to phylogenetic distances.

Information about the biological and/or biochemical properties were compiled for 26 TLPs with a complete amino acid sequence from an exhaustive survey of the literature (Additional file 8). These data were added systematically to the phylogenetic sub-trees of TLP subgroups I (Figure 5) and II (Additional file 9). Among these 26 TLPs, 21 have antifungal activity and nine have endo-β-1,3-glucanase activity. Surprisingly, antifungal TLPs are widespread among eukaryotes, as 13 are present in TLP subgroup I and 8 are in TLP subgroup II. A similar widespread assortment across TLP subgroups I and II was obtained for TLPs that exhibit endo-β-1,3-glucanase or antifreeze activities. Compared with the large amount of information available concerning asterid TLPs (many functions have been described for two TLPs of subgroup I: tobacco osmotin, Nicta-1709500, and maize zeamatin, Zeama-grmzm2g394771), there is almost no functional characterization of conifer and fungal TLPs or sTLPs. One exception is TLX1, a sTLP from wheat (Triae-110836639), which is the only sTLP characterized to date and the only TLP shown to have xylanase inhibitor activity (Additional files 7 and 8). Among poplar TLPs, only the four TLPs from the poplar clade 1 (Figure 1) belong to the eukaryote TLP subgroup I (Figure 5). Proteins from TLP subgroup II have been poorly characterized, except for the rosid-specific and tree-enriched clade, which contains several proteins with described antifungal or endo-β-1,3-glucanase activities (Additional file 9). Thirty-one poplar TLPs are distributed in TLP subgroup II, including the 11 TLPs that form the poplar TLP cluster and which are assembled in the tree-enriched clade. To our knowledge, none of the proteins from subgroup III have been characterized at the functional level so far (Additional file 10).

**Figure 6:**
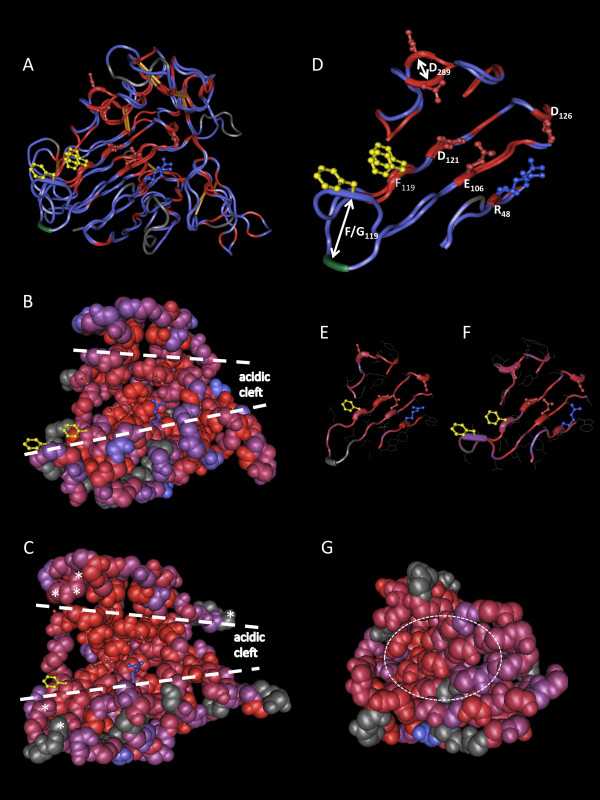
**3D structure alignment (3D-SA) and alignment mapping on 3D structure (AM-3D) of eukaryote TLPs**. Amino acids of the REDDD and FF motifs are represented with side-chains in balls and sticks. Color code of side-chains, red: negatively charged; blue: positively charged; yellow: hydrophobic. White dashed-lines indicate acidic cleft limits. (A), 3D-SA of tobacco PR-5d and cherry Pru av 2. Protein backbone color code, red: identical amino acids; blue: different amino acids; grey: unaligned residues, green: glycine/phenylalanine residues discussed in the text. Disulfide bonds are in orange. (B), AM-3D of 9 subgroup I TLPs using the PR-5d structure as template. The four-color code of the protein backbone (from red to blue) corresponds to a decrease in amino acid conservation. (C), AM-3D of 15 subgroup II TLPs using the Pru Av 2 structure as template. Color code and annotations are as in B. Amino acids under diversifying selection [39] are indicated by white asterisks. (D, E and F), Highlights of β-sheets forming the acidic cleft in A, B and C respectively. Color code is similar to that in A, B and C. In D, the residues forming the REDDD and FF motifs are numbered as in Figure 4. White arrows indicate motif differences discussed in the text. (G), AM-3D of the 9 small-TLPs indicated in Additional file 7 using the TLX1 structure as template. Color code is similar to that in B. A white dashed ellipse marks the missing acidic cleft.

To estimate how TLP structural diversity influences biological and biochemical functions, a 3D structure alignment (3D-SA) was performed with the phylogenetically most distinct TLP structures available among the seven solved to date: the tobacco PR-5d (Nicta-1709500; PDB: 1AUN) from TLP subgroup I and the cherry Pru Av 2 (Pruav-1729981; PDB: 2AHN) from TLP subgroup II (Figure 6). In general, the 3D structures of these TLPs superimpose well, especially the region forming the acidic cleft. Indeed, this region, as well as two hydrophobic or aromatic residues (generally Phe or Tyr), are important for the antifungal or lytic activities of TLPs (Figure 6, [21]). However, although well conserved, some residues of the REDDD and FF motifs adopt slightly different positions in these two TLPs. For example in the Pru Av 2 structure, the side chain of the aspartate at position 289 (D_289_) is oriented outside the acidic cleft and the phenylalanine residue F_119 _is replaced by a small non-aromatic residue (Gly) that is positioned differently. It is not clear whether these small differences have a significant impact on the substrate selectivity or protein function. Primary sequence alignment mapping on 3D structures (AM-3D) of PR-5d and Pru Av 2 with sequences from subgroups I and II, respectively, confirmed that the acidic cleft is the most conserved region among eukaryote TLPs (Figure 6). By contrast, although the REDDD amino acids are conserved in most sTLPs, AM-3D of several sTLP sequences with the recently solved structure of wheat TLX1 (PDB: 1KWN) revealed neither an acidic cleft nor any particular conserved region which could be linked to the reported xylanase inhibitor function (Figuer 6, [12]).

**Figure 7:**
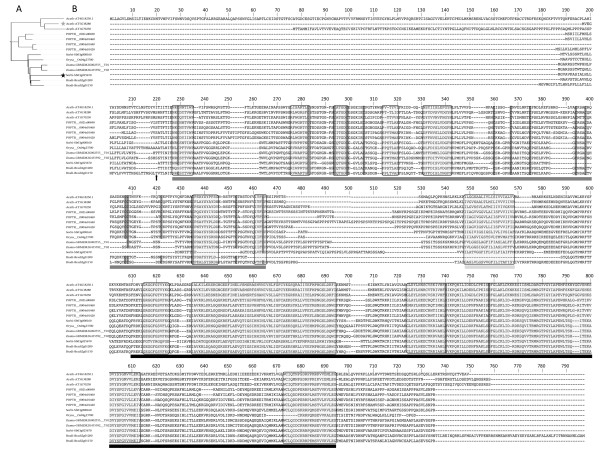
**Amino acid sequence comparison of plant TLP-kinases (TLP-Ks)**. (A), Neighbour-joining tree of the 14 TLP-Ks identified in plants. Branch lengths are proportional to phylogenetic distances. Black star: sTLP-K from *Brachypodium distachyon*; grey star: TLP-K from *Arabidopsis thaliana *described in [51]. (B), ClustalW amino acid alignment using the parameters described by [6] and manually adjusted. Thaumatin and protein kinase domains are respectively underlined in dark grey and black. Phobius [72] predicted transmembrane domain is underlined in light grey. Shaded boxes indicate highly conserved sequences. The arrow indicates the end of the predicted signal peptide. Vertical bars indicate cysteine residues in the thaumatin domain and aspartate residues forming the catalytic site of the kinase domain.

Alignment of the 14 TLP-Ks identified from six different plant species (two dicots and four monocots), including the four poplar TLP-Ks, revealed that the thaumatin domain of TLP-Ks is similar to that of typical TLPs, possessing both the conserved residues involved in the acidic cleft and the cysteine residues (Figure 7). The protein kinase domain of TLP-Ks is extremely well conserved, even among monocots and dicots, and contains two fully conserved residues D_740 _and D_758 _known to be part of the catalytic motif [33]. A predicted TM domain is present between the thaumatin and the protein kinase domains in all TLP-K sequences (Figure 7, Additional file 5), except Bradi-2g01200, which might be due to an erroneous interdomain annotation in the *Brachypodium distachyon *genome.

## Discussion

The recent release of the *P. trichocarpa *genome, the first tree genome available, paved the way for high-throughput genomic and computational analyses of multigene families, and has defined *Populus *as a model organism in forest biology [34]. Considering that leaf rust fungi are responsible for considerable damage in poplar plantations, the *Populus*/*Melampsora *interaction has emerged as a model pathosystem in forest pathology [26]. In order to decipher the molecular basis of poplar resistance against this biotrophic fungus, in-depth and exhaustive studies of defense-related functions require a reliable annotation of gene families before we can understand their structural and functional diversity. We have therefore performed a genome-wide analysis of the TLP multigene family, which comprises many stress-inducible proteins in *P. trichocarpa. *Extension of the phylogenetic analysis to include TLPs from other eukaryotes extends our knowledge about the evolution of TLPs.

### TLPs in plant genomes: an evolutionary diversification

The history of TLP multigene families retraced from 18 plant genome sequences shows a strong evolutionary diversification from the green alga *C. reinhardtii *to the black cottonwood tree *P. trichocarpa *(Table 1). In the co-evolution of host-microbe interactions, plants and fungi acquire new weapons that promote their resistance or virulence, respectively [35]. As a consequence of this arms race, the size of some multigene families involved in resistance (such as NB-LRRs) has greatly increased in poplar and other higher plants [36]. It is accepted that this increase, sometimes specific to certain organisms, represents an important means for generating functional diversity via sub- or neo-functionalization of paralogs [37]. Analysis of natural selection is increasingly used in plant pathology to estimate how evolutionary forces impact genes and corresponding proteins, from the scale of amino acid sites to gene families [38]. Poplar TLP genes have recently been investigated for natural selection. The four TLP-Ks and 10 of the 11 TLPs that belong to clade 2 (i.e. the poplar TLP cluster) were shown to be driven by diversifying (positive) selection (Figure 1, [39]). More precisely, several exposed amino acids of TLPs are under diversifying selection, whereas amino acids forming the acidic cleft are under purifying (negative) selection and thus well-conserved (Figure 6). Conservation of the acidic cleft could be necessary to maintain antifungal activity, whereas diversification of exposed amino acids could avoid recognition by pathogen enzyme-inhibitors or proteases [40].

### Is the antifungal activity of TLPs a universal property?

Historically, TLPs have been described as biotic and abiotic stress-responsive proteins and were called TLPs/PR5 or osmotin/osmotin-like proteins (OLPs), depending on the stress condition (i.e. biotic or abiotic stress, respectively) in which these proteins or their closest homologs were first described. As already suggested by Shatters and collaborators [6], phylogenetic analyses do not support this separate nomenclature that generates semantic confusion in the literature [41]. Our broad sequence analysis of eukaryote TLPs confirms that there is no clear difference among TLPs and OLPs, since different TLP functions are not separated by distinct phylogenetic clades. In fact, the major biochemical properties of TLPs such as antifungal or endo-β-1,3-glucanase activity are widespread among eukaryotes (Figure 5 and Additional file 9). At the structural level, most TLPs are predicted to share a conserved acidic cleft, which is usually associated with an antifungal property, suggesting that this property is universal among eukaryote TLPs (Figure 6). Although more subtle conformational differences might explain the large variety of properties described so far for TLPs, the phylogenetic and functional data do not justify adoption of a distinct nomenclature between biotic- and abiotic-responsive TLPs. An exception to this statement might be considered for TLPs with different domain organisations such as sTLPs or TLP-Ks. Indeed, sTLPs are assumed to act as xylanase inhibitors and no antifungal activity has yet been reported [12]. This functional divergence is consistent with the important structural differences observed and in particular with the absence of a well-defined acidic cleft (Figure 6).

### Poplar TLPs: stress-responsive proteins, but not only

The release of the *P. trichocarpa *genome version 2.0 and its integration into the Phytozome portal enabled a drastic improvement of TLP gene annotation and the validation of more than 70% of the 59 *TLP *gene models from the *Populus *genome version 1.1 [Additional file 1, [24]). The expression analysis of TLPs during biotic and abiotic stresses supports the idea that TLPs, like other PR proteins, belong to a general plant stress response pathway rather than being specific to distinct stresses, as often hypothesized [1]. This is exemplified by *PopTLP1*, whose expression is induced by diverse environmental constraints such as high ozone, UV-B, drought, copper and infection by rust fungi (Figure 1 and Additional file 4). In addition, *PopTLP1 *is the closest homolog of *A. thaliana Atosm34 *(At4g11650), which also accumulates during both biotic and abiotic stress conditions [8,42]. The RT-qPCR expression profile of *PopTLP1 *in rust-infected poplar leaves confirmed transient transcript accumulation during host-specific resistance (Additional file 4). This profile is in accordance with results obtained by similar approaches for several PR proteins in this pathosystem [27] and confirms the involvement of TLPs in poplar defense.

However, the fact that only 19 of the 42 poplar *TLPs *are transcriptionally regulated in the stress conditions investigated suggests that their role in poplar might not be restricted to stress response but that they could have other roles, such as during development. Indeed, some TLPs have been reported to accumulate during plant developmental stages or conditions such as in ovular secretions or during leaf aging [43,44]. In addition, TLPs have been extensively described as ripening-associated proteins that accumulate strongly in fruit during maturation [45,46]. It has been shown recently in hybrid poplars that two TLPs belonging to the tree-specific and stress-responsive TLP cluster (Figure 1 and 2) are present in the phloem of healthy non-stressed plants [47]. Taken together, these results suggest that the expansion of this multigene family in poplar could also be related to tree-specific developmental stages.

### The poplar TLP cluster contains tree-specific and stress-responsive proteins

The poplar TLP cluster is an assembly of 11 successive genes on scaffold 1 and is considerably enriched in TE for a gene-containing genomic region. Indeed, TE account for 52% of the TLP cluster region compared with an average coverage of 42% in the whole genome [24]. Moreover, LTR TE from the Gypsy class are specifically over-represented, covering 37% of the TLP cluster compared with 5% in the whole *P. trichocarpa *genome sequence (Figure 2, [24]). This class of TE might be a source of genome plasticity in plants [48]. The very well-conserved exon-intron structure of the genes in the TLP cluster supports a mechanism of tandem duplication from a unique ancestral gene (Figure 1). Taken together, these results strongly suggest that this cluster likely resulted from recent tandem duplications driven by TE activity. Futhermore, the TLP cluster appears to be highly responsive to fungal infection in poplar and belongs to a rosid-specific and tree-enriched clade in our complete phylogeny of eukaryote TLPs (Figure 1, Figure 3 and Additional file 9). TLPs from cherry, chestnut, apple and peach trees that exhibit antifungal and/or endo-β-1,3-glucanase activities (Additional file 8[49,50]) also belong to this clade. Thus, the TLP cluster appears to be a tree-specific and rust-responsive group of TLPs that are of outstanding interest for further analyses focusing on tree and TLP specificities in defense against pathogens. More precisely, two TLPs recently identified at the protein level in the phloem of hybrid poplar constitute excellent candidates for future investigations ([47], Figure 1).

### TLP-Ks: defense proteins recruited for signaling?

TLP-Ks result from the fusion between two genes coding for a TLP and a protein kinase. They have been hypothesized to act as receptor-like kinases (RLKs), where the extracellular TLP could sense pathogens and the cytoplasmic kinase could relay downstream signaling [23]. This assumption was strengthened by the demonstration that plants overexpressing an *A. thaliana *TLP-K showed a delay in the appearance of disease symptoms [51]. The ability of plants to recruit defense proteins to form a RLK involved in pathogen sensing has already been suggested for PR1 and PR3 [1]. The strong homology of the kinase domain between TLP-K and some lectin-kinases reinforces the speculation about the potential role of TLP-K in the induction of the defense system, since a rice lectin-kinase has been shown to confer resistance to the rice blast [52]. In the *P. trichocarpa *genome, three TLP-Ks are organized in tandem on scaffold 4 and are interspersed by other protein kinase-encoding genes. This genomic region is referred to as the 'TLP-K cluster' (Figure 2). Genetic and physical mapping of *Melampsora *rust resistance genes in natural populations of *P. trichocarpa *identified a locus encoding two *TLP-K *genes on chromosome 4, which possibly corresponds to the TLP-K cluster in scaffold 4 of the *P. trichocarpa *genome sequence [53]. Hence, although evidence is still needed to clarify the exact role of these TLP-Ks in poplar, this opens interesting perspectives concerning new RLK types related to poplar defense against *Melampsora *spp. rust pathogens.

## Conclusion

TLPs are eukaryote proteins that constitute small and monophyletic families in invertebrate animals and fungi whereas they are more diverse and are organized in large multigene families in plants. Regardless of their origin, it appears that many typical TLPs possess an antifungal activity, which is probably linked to a conserved acidic cleft in their 3D structure. In plants, TLPs have undergone a drastic evolutionary diversification including the evolution of tree-enriched clades and of TLPs fused to protein kinase domains. The poplar genome encodes 42 validated *TLP *gene models, including four TLP-kinases. Some poplar TLP transcripts accumulate specifically under abiotic or biotic stress conditions, which can be strongly correlated with their phylogeny. In the poplar genome, a tree-specific and stress-responsive cluster of tandemly-duplicated *TLP *genes should be of interest for understanding the unique attributes of defense against pathogen attacks that have evolved in trees.

## Methods

### Identification and annotation of TLP genes in P. trichocarpa

TLP genes were identified in the *P. trichocarpa *'Nisqually-1' genome using thaumatin and osmotin keywords and amino acid sequence homology searches. Manual gene annotation was performed by finding missing start/stop codons, by defining correct exon/intron borders, by analyzing perfectly matching ESTs and by taking into account the amino acid conservation of the thaumatin domain (Pfam: PF00314) and in particular the position of conserved cysteines. Alignments with closest homologs in the Phytozome portal were used to reconstruct gene structure and corresponding amino acid sequences. Allelic versions detected by the Phytozome annotation (two adjacent genes in the genome assembly v1.1 that correspond to a single locus in the v2.0 assembly) or incomplete genes were discarded and not considered for further analysis. Transposable element analysis was carried out with the CENSOR software available on the Giri database [54].

### Search for TLP in public genomic databases and sequence analyses

The 18 plant genome sequences available on the Phytozome portal [29] (June 2010) were mined using sequence homology searches. Only *TLP *gene models encoding a complete TLP domain were reserved for sequence comparison. The NCBI protein database [32] was mined using sequence homology and keyword searches (i.e. thaumatin and osmotin). TLP sequences from non-sequenced plants, fungi and animals were individually examined and amino acid sequences with a complete thaumatin family domain were retained for further analyses. Sequences from *M. larici-populina *and *P. graminis *f.sp. *tritici *were retrieved from the Joint Genome Institue (JGI, [55]) and the Broad Institute [56] websites, respectively.

### Sequence alignment and construction of phylogenetic tree

For all amino acid sequence comparisons, the thaumatin domain covering almost 95% of the mature TLP protein was considered. Limits of the TLP domain were defined as N-x-C-x(3)-V/I-W and Y-x-I/V-x-F-C-x in the N- and C-terminal ends, respectively. Amino acid sequence alignments were performed using ClustalW as described by [6], DIALIGN (http://dialign-tx.gobics.de/) as well as MAFFT (http://mafft.cbrc.jp/alignment/server/) methods. In all cases, the raw output alignments required deep manual re-adjustment to proceed further with phylogenetic reconstruction. Raw alignments were thus imported into the Molecular Evolutionary Genetics Analysis (MEGA) package 4.1 [57] and manually adjusted. Phylogenetic analyses were conducted using the Neighbour-Joining method with the pairwise deletion option for handling alignment gaps and the Poisson correction model for distance computation. Bootstrap tests were conducted using 1,000 replicates. Branch lengths are proportional to phylogenetic distances.

### Expression of poplar TLPs

Transcriptional data for *Populus*/*Melampsora *interaction were extracted from published studies with significant fold-changes as described by the respective authors (Figure 1, [27,30,31]). Other information pertaining to the poplar transcriptome during stress-related situations were extracted from the PopGenIE portal [58,59] and from the literature. Proteomic data for *Populus/Melampsora *interaction were retrieved from the PROTICdb database [60,61] and from the literature for other stress-related situations.

### 3D structure analyses

3D-SA and MA-3D were carried out with Cn3D software [62]. Sequences were manually aligned using the integrated sequence viewer in editor mode and reference structures were retrieved from NCBI structure database [32]. For MA-3D, sequence conservation has been estimated with a four level color code from red to blue, reporting a respectively strong to weak amino acid variety.

### RT-qPCR analyses

Isolates 98AG31 (virulent, pathotype 3-4-7) and 93ID6 (avirulent, pathotype 3-4) of *M. larici-populina *were used in this study. Rust urediniospore multiplication and plant inoculation procedures were performed as previously described [27], using the same inoculum doses (100,000 urediniospores/ml), leaf plastochron indexes for detached *P. trichocarpa *X *Populus deltoides *'Beaupré' leaves and identical culture conditions. For time-course infection analyses, leaves were harvested at the following time-points: 0, 12, 15, 18, 21, 24, 36, 48, 72, 96, 120 and 168 hpi. RNA extraction, quality control and cDNA synthesis were performed as previously described in [27]. In order to assess transcript levels by RT-qPCR, specific primers for the *PopTLP1 *gene (Poptr_0001s09570 in *P. trichocarpa *genome; 5' CCAGACTTGGTATCTTAATG; 3' GTTACCAAACTGATTTAACG) were designed and quantitative PCR was carried out as previously described [63], with technical and biological duplicates. Transcript expression was normalized to a reference ubiquitin transcript (Poptr_0015s01600 in *P. trichocarpa *genome; 5' GCAGGGAAACAGTGAGGAAGG; 3' TGGACTCACGAGGACAG) using ratio calculation as described in [64].

## Abbreviations

TLP: thaumatin-like protein; PR: pathogenesis-related; GPCR: G protein-coupled receptor; sTLP: small-TLP; TM: transmembrane domain; TLP-K: TLP-kinase; CDS: coding DNA sequence; sTLP-K: small-TLP-kinase; TE: transposable element; 3D-SA: 3D structure alignment; AM-3D: alignment mapping on 3D structure; OLP: osmotin-like protein; JGI: joint genome institute; RLK; receptor-like kinase; EST: expressed sequence tag; hpi: hour-post inoculation; NB-LRR: nucleotide binding-leucine rich repeat; LTR: long terminal repeat.

## Authors' contributions

BP and SD performed conceptual and experimental designs. BP carried out experimental procedures, *in silico *analyses and drafted the manuscript. IM compiled transcriptional data concerning *Populus-Melampsora *interactions from the literature. SD and NR supervised the work and helped with conceptual design and data analysis. All authors participated in depth reading and revising the manuscript. All authors read and approved the final manuscript.

## Supplementary Material

Additional file 1 **Annotation of *TLP *genes in the *Populus trichocarpa *'Nisqually-1' genome sequence**. ^**a**^*TLP *gene models retrieved in the *Populus trichocarpa *'Nisqually-1' genome version 1.1 from the JGI website [55]. ^**b**^*TLP *gene models retrieved in the *P. trichocarpa *'Nisqually-1' genome version 2.0 from the Phytozome portal [29]. Click here for file

Additional file 2 **List of amino acid sequences deduced from the 42 *P. trichocarpa TLP *gene models**. Stop codons are represented by asterisks. Click here for file

Additional file 3 **Transposable element (TE) features of the TLP cluster**. ^a^Percentage of the 350 kb total length of the TLP cluster covered by TE. Click here for file

Additional file 4 ***PopTLP1 *RTqPCR expression profile**. Total RNA was isolated from mock-inoculated or inoculated leaves of *Populus trichocarpa X Populus deltoides *'Beaupré' with either compatible (white diamonds, strain 98AG31) or incompatible (black diamonds, strain 93ID6) strains of *Melampsora larici-populina *between 12 and 168 hours post-inoculation (hpi). RT-qPCR results are presented as expression ratios. *Populus *ubiquitin transcripts were as a reference gene for normalization. n = 2 (except for I168, n = 1), error bar: standard deviation. Click here for file

Additional file 5 **Small-TLP-kinase domains and features**. The signal peptide and the transmembrane domain of the small-TLP-kinase of *Sorghum bicolor *(Sb03g025670) are predicted by the Phobius program [72]. Click here for file

Additional file 6 **Protein accession numbers and phylogenetic classification of corresponding species used in this study**. ^**a**^Organism code used in the study, corresponding to the 3 first letters of the genus name followed by the 2 first letters of the species name (ex.: *Arabidopsis thaliana *= Arath), except for *Nepenthes species pluralis *(Nep.spp) and *Melampsora larici-populina *(Mel.la.po). ^**b**^Number of TLP sequences used in this study. ^**C**^Accession numbers are preceded by the organism code. Sequences retrieved from the NCBI protein database [32] are in black, those retrieved from the Phytozome portal [29] are in red. The 3 sequences of *M. larici-populina *were retrieved from the JGI genome website [55] and are labelled with JGI protein IDs. Click here for file

Additional file 7 **Neighbour-joining tree of eukaryote small-TLPs**. Branch lengths are proportional to phylogenetic distances. Branch color and protein ID codes correspond to those in Figures 3 and 5, respectively. Supplemental sequences from the *Puccinia graminis *f.sp. *tritici *genome sequence were retrieved from the Broad Institute website [56] (gene IDs PGTG_00965.2; PGTG_00963.2; PGTG_19613.2; PGTG_19646.2). Black star: small-TLP-Kinase from *Sorghum bicolor*; grey star: TLX 1 from *Triticum aestivum*; black arrows: sequences used for the structural analysis in Figure 6. Click here for file

Additional file 8 **List of functionally characterized TLPs described in this study**. In some cases, several studies participated to the characterization of a given TLP function. For clarity, only one reference is given per function and per protein (most relevant, otherwise first published). ^**a**^GPCRs: G-Protein-Coupled Receptors. ^**b**^Numbers refer to the complete reference in the text. Click here for file

Additional file 9 **Neighbour-joining tree of the 341 thaumatin domains of TLP Subgroup II**. Functionally characterized TLPs and corresponding functions are indicated. Poplar sequence names are in red. The five-letter code before proteins IDs indicate genus and species. Red arrows indicate protein structures used for 3D structure alignment while black arrows indicate sequences used for alignment mapping on 3D structure in Figure 6. Antifungal column includes both *in vitro*- and transgenic-based antifungal demonstrations. In the other column, a: transgenic abiotic stress protection; b: antifreeze activity; c: membrane permeabilization activity; d: xylanase inhibitor; e: α-amylase/trypsin inhibition; f: apoptosis-inducing in yeast; g: GPCR binding; h: CMV1-a binding; i: glycoprotein binding; j: endo-β-1,3-glucanase activity; k: 3D structure solved. References corresponding to these data are summarized in Additional file 8. Branch lengths are proportional to phylogenetic distances. Click here for file

Additional file 10 **Neighbour-joining tree of uncharacterized eukaryote TLPs from TLP subgroup III**. Branch lengths are proportional to phylogenetic distances. Branch color and protein IDs codes correspond to those in Figures 3 and 5, respectively. Poplar sequence names are in red. Click here for file

## Ackowledgements

We warmly thank our colleagues Francis Martin, Annegret Kohler and Pascal Frey at INRA Nancy for regular and fruitful discussions about the poplar/rust pathosystem and gene family annotation in the poplar genome. We also thank Christine Delaruelle and Patrice Vion for great technical help during RNA extraction and poplar culture respectively, Claude Murat for valuable advice on transposable elements and phylogenetic analyses, Bénédicte Favre for *M. larici-populina *spore conservation and Stéphane Hacquard for very helpful discussions on genome annotation and quantitative PCR. This work was funded by the 'Institut National de la Recherche Agronomique', 'Région Lorraine' and support grants to Sébastien Duplessis and Nicolas Rouhier.
