# Supplementary material for: Genome-wide analysis of eukaryote thaumatin-like proteins (TLPs) with an emphasis on poplar
Source: BMC Plant Biol. 2011 Feb 15;11:33. doi: 10.1186/1471-2229-11-33 (PMC3048497; doi:10.1186/1471-2229-11-33)
Supplement: Additional file 1 — Annotation of TLP genes in the Populus trichocarpa 'Nisqually-1' genome sequence. aTLP gene models retrieved in the Populus trichocarpa 'Nisqually-1' genome version 1.1 from the JGI website [55]. bTLP gene models retrieved in the P. trichocarpa 'Nisqually-1' genome version 2.0 from the Phytozome portal [29]. [file 1471-2229-11-33-S1.PDF]

gene model in genome version 1.1 (JGI)[a]	gene model in genome version 2.0 (Phytozome)[b]	Annotation status
eugene3.00010939	POPTR_0001s09000	Validated
gw1.I.9073.1	POPTR_0001s09030	Validated
gw1.I.8918.1	POPTR_0001s09570	Validated
fgenesh4_pm.C_LG_I000505	POPTR_0001s21790	Validated
estExt_fgenesh4_pm.C_LG_I0604	POPTR_0001s22810	Validated
grail3.0149000101	POPTR_0001s22830	Validated
eugene3.01490008	POPTR_0001s22850	Validated
gw1.149.43.1	POPTR_0001s22860	Validated
fgenesh4_pg.C_scaffold_149000012	POPTR_0001s22870	Validated
estExt_fgenesh4_pg.C_6010001	POPTR_0001s22880	Validated
eugene3.01490017	POPTR_0001s22900	Validated
grail3.0149000701	POPTR_0001s22910	Validated
fgenesh4_pg.C_scaffold_149000024	POPTR_0001s22920	Validated
fgenesh4_pg.C_scaffold_149000025	POPTR_0001s22930	Validated
fgenesh4_pg.C_scaffold_149000029	POPTR_0001s22960	Validated
estExt_Genewise1_v1.C_LG_I1344	POPTR_0001s24430	Validated
gw1.II.2149.1	POPTR_0002s02190	Validated
estExt_Genewise1_v1.C_LG_II2087	POPTR_0002s02200	Validated
gw1.II.1823.1	POPTR_0002s08760	Validated
gw1.III.1427.1	POPTR_0003s01430	Validated
gw1.IV.1622.1	POPTR_0004s01420	Validated
gw1.7884.1.1	POPTR_0004s01440	Validated
gw1.IV.1660.1	POPTR_0004s01460	Validated
fgenesh4_pm.C_LG_IV000530	POPTR_0004s17890	Validated
fgenesh4_pg.C_scaffold_57000074	POPTR_0005s11480	Validated
gw1.57.277.1	POPTR_0005s11490	Validated
gw1.V.5175.1	POPTR_0005s16030	Validated
gw1.V.1814.1	POPTR_0005s26240	Validated
fgenesh4_pm.C_LG_V000656	POPTR_0005s26250	Validated
gw1.VI.993.1	POPTR_0006s08840	Validated
estExt_fgenesh4_kg.C_LG_IX0046	POPTR_0009s03410	Validated
gw1.IX.1273.1	POPTR_0009s13480	Validated
estExt_Genewise1_v1.C_LG_IX1261	POPTR_0009s13510	Validated
gw1.X.2095.1	POPTR_0010s20840	Validated
fgenesh4_pg.C_LG_XII000356	POPTR_0012s00430	Validated
estExt_Genewise1_v1.C_LG_XII1843	POPTR_0012s04520	Validated
gw1.40.444.1	POPTR_0014s04020	Validated
gw1.XV.22.1	POPTR_0015s00310	Validated
gw1.XV.1016.1	POPTR_0015s04510	Validated
gw1.XVII.817.1	POPTR_0017s11190	Validated
grail3.0020019002	POPTR_0018s10490	Validated
gw1.XI.1265.1	POPTR_0021s00690	Validated
estExt_Genewise1_v1.C_4720001	POPTR_0001s09000	Unvalidated / allele of POPTR_0001s09000
gw1.6840.1.1	POPTR_0001s09570	Unvalidated / allele of POPTR_0001s09570
gw1.7217.6.1	POPTR_0001s21790	Unvalidated / allele of POPTR_0001s21790
grail3.0357000301	POPTR_0001s22850	Unvalidated / allele of POPTR_0001s22850
eugene3.17380001	POPTR_0001s22960	Unvalidated / allele of POPTR_0001s22960
gw1.1610.7.1	POPTR_0002s08760	Unvalidated / allele of POPTR_0002s08760
gw1.II.1309.1	POPTR_0002s13430	Unvalidated / allele of POPTR_0002s13430
gw1.1112.9.1	POPTR_0004s01420	Unvalidated / allele of POPTR_0004s01420
gw1.IV.1641.1	POPTR_0004s01420	Unvalidated / allele of POPTR_0004s01420
eugene3.11120001	POPTR_0004s01440	Unvalidated / allele of POPTR_0004s01440
gw1.5724.7.1	POPTR_0004s01440	Unvalidated / allele of POPTR_0004s01440
eugene3.00041331	POPTR_0004s17880	Unvalidated / incomplete
gw1.XI.1274.1	POPTR_0021s00670	Unvalidated / incomplete
gw1.IV.1632.1	POPTR_0004s01440	Unvalidated / incomplete
gw1.171.48.1	no corresponding model gene	Unvalidated / incomplete
gw1.357.4.1	POPTR_0001s22860	Unvalidated / incomplete
eugene3.00010938	no corresponding model gene	Unvalidated / incomplete
