# Supplementary material for: Genome-wide analysis of eukaryote thaumatin-like proteins (TLPs) with an emphasis on poplar
Source: BMC Plant Biol. 2011 Feb 15;11:33. doi: 10.1186/1471-2229-11-33 (PMC3048497; doi:10.1186/1471-2229-11-33)
Supplement: Additional file 3 — Transposable element (TE) features of the TLP cluster. aPercentage of the 350 kb total length of the TLP cluster covered by TE. [file 1471-2229-11-33-S3.PDF]

repeat class	Nb of fragments	covered length (kb)	[a]covered length (%)
Endogenous Retrovirus	7	<1	<1
Non-LTR Retrotransposon	40	6	2
DNA transposon	169	31	8
LTR Retrotransposon	345	170	43
LTR-Gypsy	285	147	37
LTR-Copia	60	23	6
Total Transposable Element	561	208	52
