# Supplementary figures and images for: Genome-wide analysis of eukaryote thaumatin-like proteins (TLPs) with an emphasis on poplar
Source: BMC Plant Biol. 2011 Feb 15;11:33. doi: 10.1186/1471-2229-11-33 (PMC3048497; doi:10.1186/1471-2229-11-33)

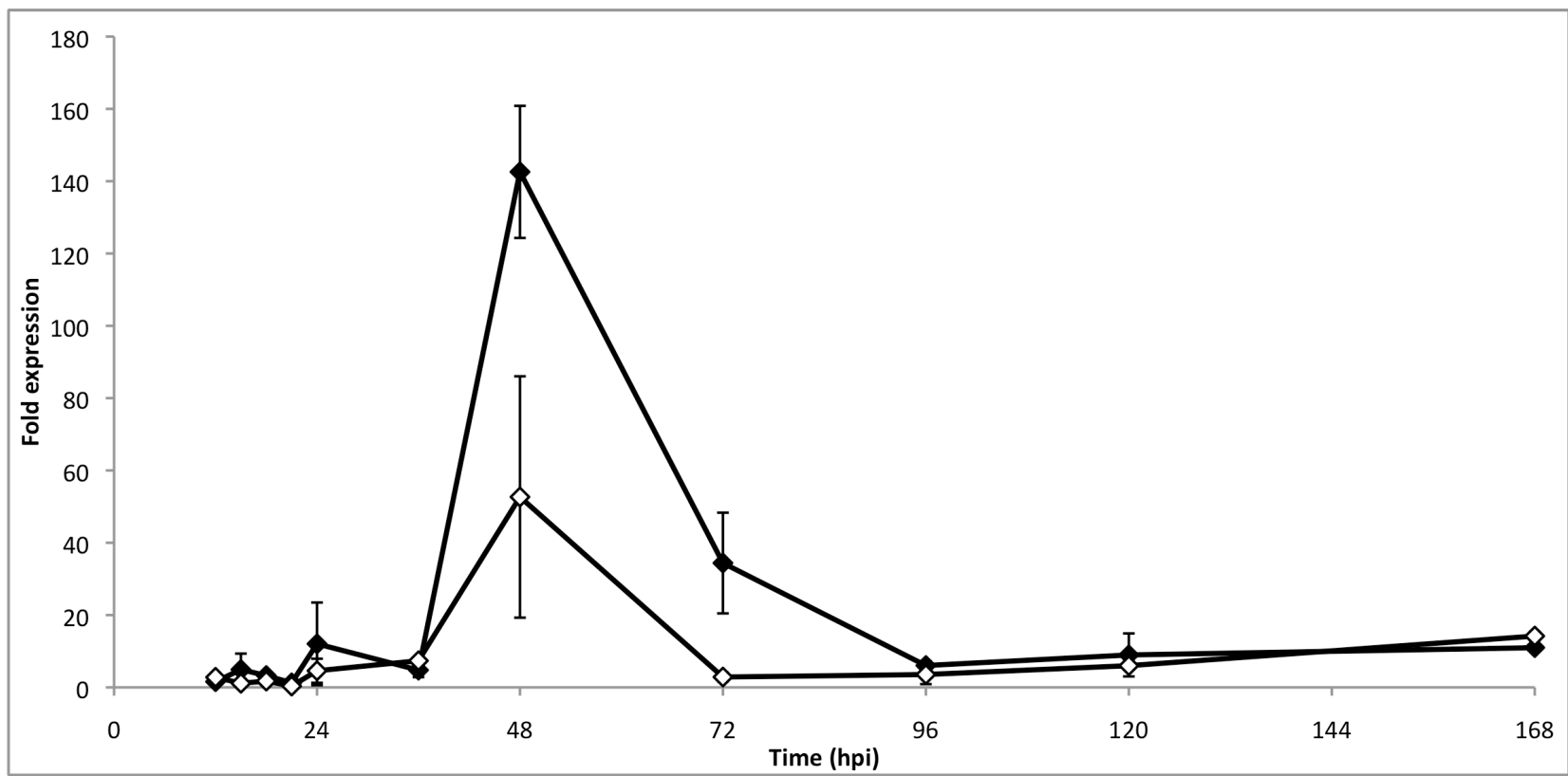

Supplement: Additional file 4 — PopTLP1 RTqPCR expression profile. Total RNA was isolated from mock-inoculated or inoculated leaves of Populus trichocarpa X Populus deltoides 'Beaupré' with either compatible (white diamonds, strain 98AG31) or incompatible (black diamonds, strain 93ID6) strains of Melampsora larici-populina between 12 and 168 hours post-inoculation (hpi). RT-qPCR results are presented as expression ratios. Populus ubiquitin transcripts were as a reference gene for normalization. n = 2 (except for I168, n = 1), error bar: standard deviation. [file 1471-2229-11-33-S4.PDF]

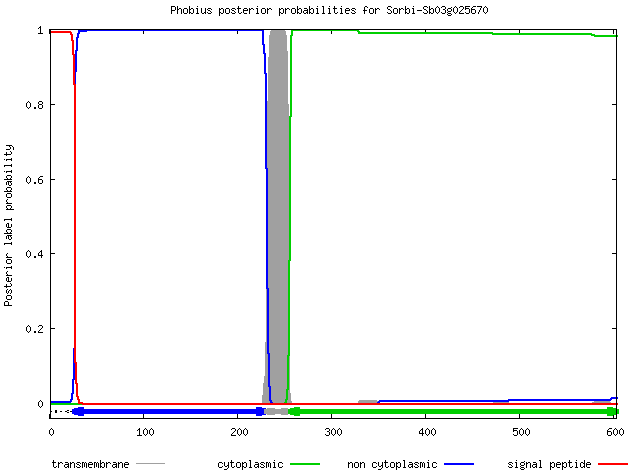

Supplement: Additional file 5 — Small-TLP-kinase domains and features. The signal peptide and the transmembrane domain of the small-TLP-kinase of Sorghum bicolor (Sb03g025670) are predicted by the Phobius program [72]. [file 1471-2229-11-33-S5.PNG]

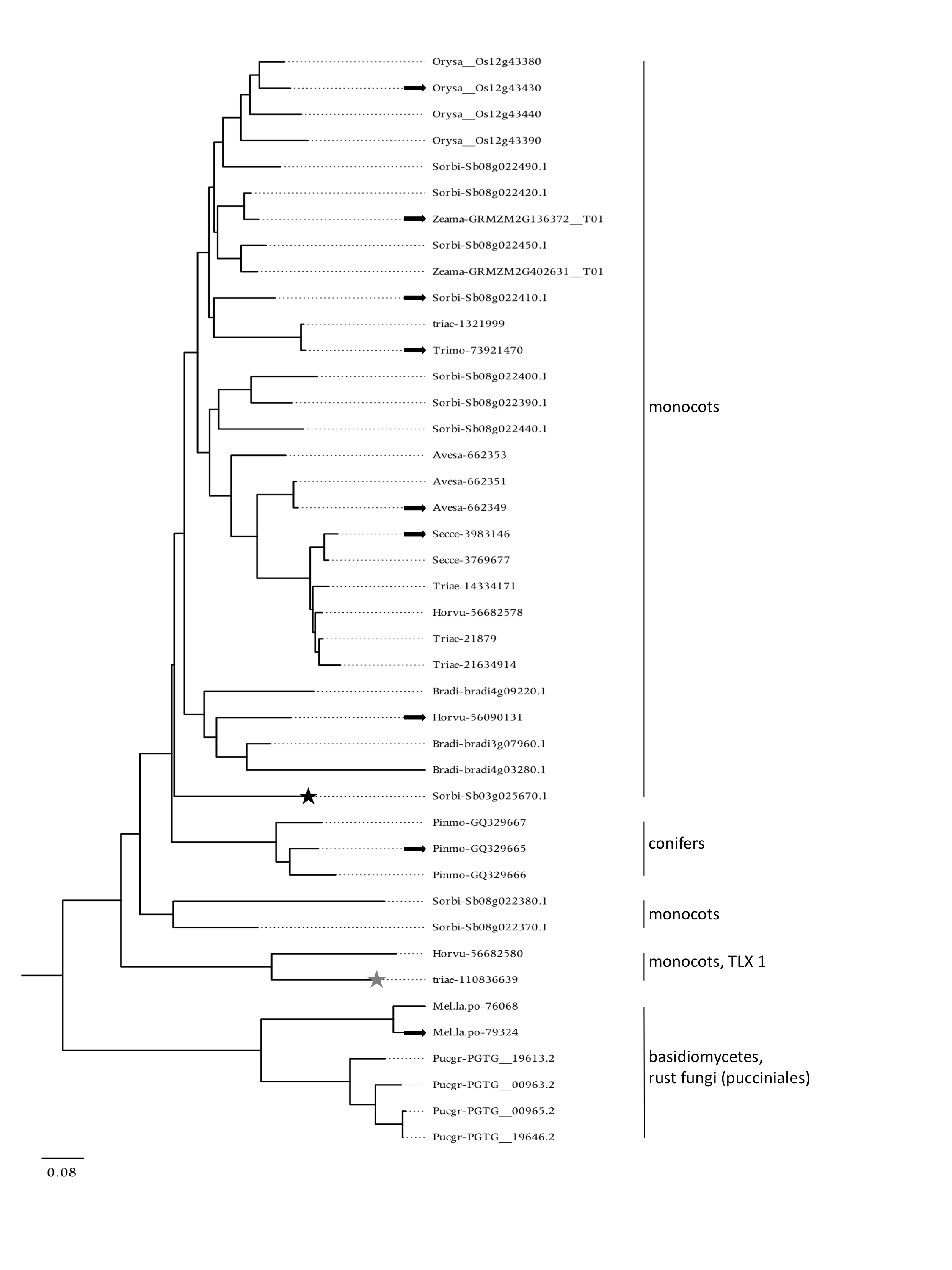

Supplement: Additional file 7 — Neighbour-joining tree of eukaryote small-TLPs. Branch lengths are proportional to phylogenetic distances. Branch color and protein ID codes correspond to those in Figures 3 and 5, respectively. Supplemental sequences from the Puccinia graminis f.sp. tritici genome sequence were retrieved from the Broad Institute website [56] (gene IDs PGTG_00965.2; PGTG_00963.2; PGTG_19613.2; PGTG_19646.2). Black star: small-TLP-Kinase from Sorghum bicolor; grey star: TLX 1 from Triticum aestivum; black arrows: sequences used for the structural analysis in Figure 6. [file 1471-2229-11-33-S7.PNG]

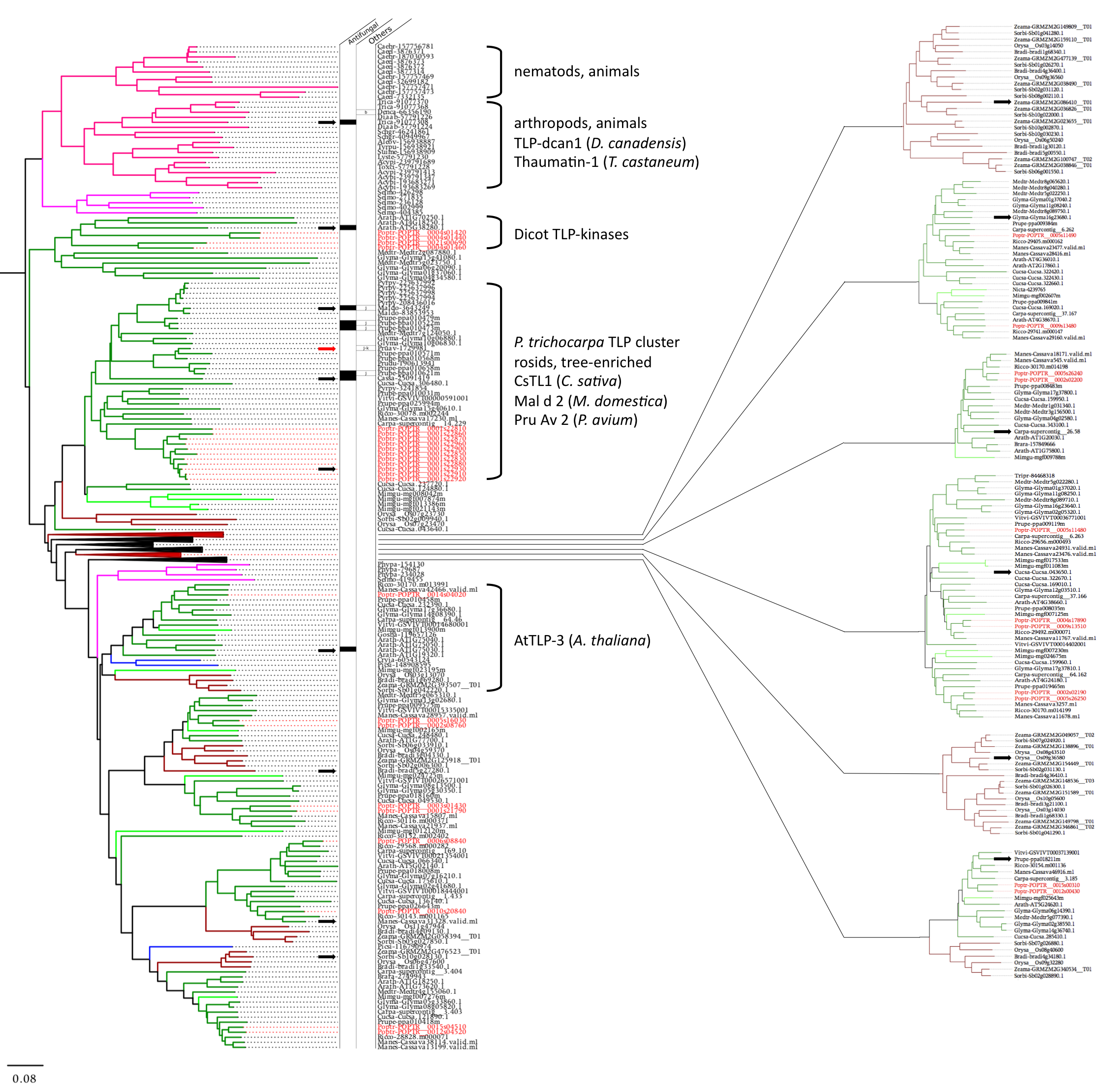

Supplement: Additional file 9 — Neighbour-joining tree of the 341 thaumatin domains of TLP Subgroup II. Functionally characterized TLPs and corresponding functions are indicated. Poplar sequence names are in red. The five-letter code before proteins IDs indicate genus and species. Red arrows indicate protein structures used for 3D structure alignment while black arrows indicate sequences used for alignment mapping on 3D structure in Figure 6. Antifungal column includes both in vitro- and transgenic-based antifungal demonstrations. In the other column, a: transgenic abiotic stress protection; b: antifreeze activity; c: membrane permeabilization activity; d: xylanase inhibitor; e: α-amylase/trypsin inhibition; f: apoptosis-inducing in yeast; g: GPCR binding; h: CMV1-a binding; i: glycoprotein binding; j: endo-β-1,3-glucanase activity; k: 3D structure solved. References corresponding to these data are summarized in Additional file 8. Branch lengths are proportional to phylogenetic distances. [file 1471-2229-11-33-S9.PNG]

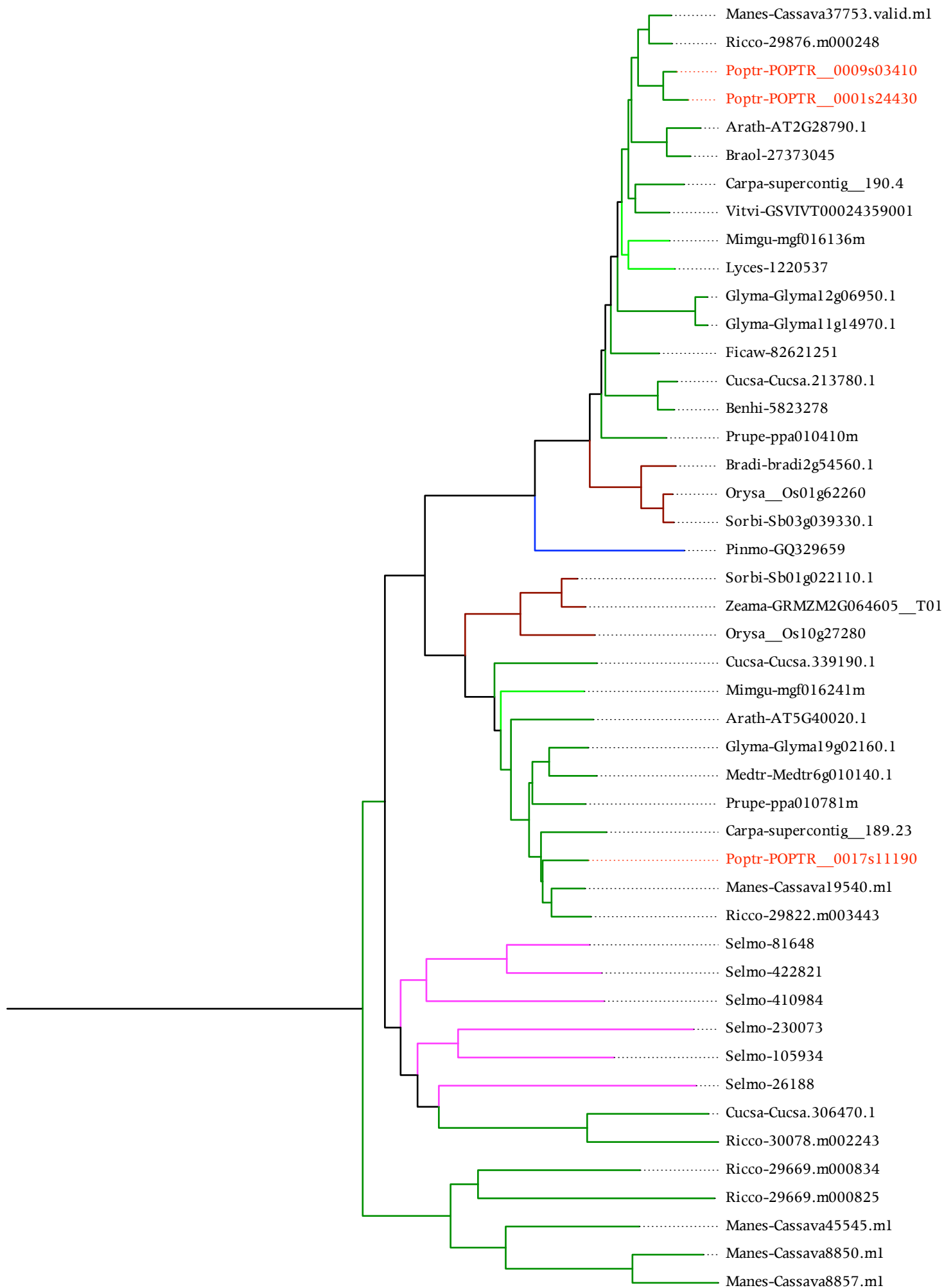

0.08

Supplement: Additional file 10 — Neighbour-joining tree of uncharacterized eukaryote TLPs from TLP subgroup III. Branch lengths are proportional to phylogenetic distances. Branch color and protein IDs codes correspond to those in Figures 3 and 5, respectively. Poplar sequence names are in red. [file 1471-2229-11-33-S10.PDF]
