# Supplementary material for: Genome-wide analysis of eukaryote thaumatin-like proteins (TLPs) with an emphasis on poplar
Source: BMC Plant Biol. 2011 Feb 15;11:33. doi: 10.1186/1471-2229-11-33 (PMC3048497; doi:10.1186/1471-2229-11-33)
Supplement: Additional file 8 — List of functionally characterized TLPs described in this study. In some cases, several studies participated to the characterization of a given TLP function. For clarity, only one reference is given per function and per protein (most relevant, otherwise first published). aGPCRs: G-Protein-Coupled Receptors. bNumbers refer to the complete reference in the text. [file 1471-2229-11-33-S8.PDF]

protein reference	protein name	species	function	reference	Ref. No.[b]
Arath-AT1G75030.1	ATLP-3	*Arabidopsis thaliana*	In vitro antifungal activity	Hu and Reddy, 1997	[73]
Arath-AT5G38280.1	PR5K	*Arabidopsis thaliana*	Transgenic antifungal protection	Guo et al., 2003	[49]
Cassa-25091419	CsTL1	*Castaena sativa*	In vitro antifungal activity	Garcia-Casado et al., 2000	[74]
Denca-66356190	TLP-*Dcan*1	*Dendroides canadensis*	Antifreeze activity	Wang and Duman, 2006	[75]
Lened-108860317	TLG1	*Lentinula edodes*	Endo-β-1,3-glucanase activity	Sakamoto et al., 2006	[5]
Maldo-3643249	Mal d 2	*Malus domestica*	In vitro antifungal activity	Krebitz et al., 2003	[76]
			Endo-β-1,3-glucanase activity	Menu-Bouaouiche et al., 2003	[48]
Musac-88191901	Ban-TLP	*Musa acuminata*	Endo-β-1,3-glucanase activity	Barre et al., 2000	[77]
			In vitro antifungal activity	Menu-Bouaouiche et al., 2003	[48]
			Tridimensionnal structure	Leone et al., 2006	[78]
Nicta-1709500	Osmotin	*Nicotiana tabacum*	Endo-β-1,3-glucanase activity	Grenier et al., 1999	[79]
			In vitro antifungal activity	Woloshuk et al., 1991	[80]
			Apoptosis causing in Yeast	Narasimhan et al., 2001	[81]
			Transgenic abiotic stress protection	D'Angeli and Altamura, 2007	[82]
			Membrane permeabilization	Abad et al., 1996	[83]
			Glycoproteins binding	Ibeas et al., 2001	[84]
			GPCRs[a] binding	Narasimhan et al., 2005	[14]
			Tridimensionnal structure	Min et al., 2004	[85]
Nicta-46250717	PR-5d	*Nicotiana tabacum*	In vitro antifungal activity	Koiwa et al., 1997	[86]
			Tridimensionnal structure	Koiwa et al., 1999	[21]
Orysa-LOC_Os12g43450.1	D34	*Oryza sativa*	Transgenic antifungal protection	Velazhahan and Muthukrishnan, 2003	[87]
Pruav-1729981	Pru Av 2	*Prunus avium*	Endo-β-1,3-glucanase activity	Grenier et al., 1999	[79]
			Tridimensionnal structure	Dall'Antonia et al., 2005	[88]
Prupe-ppa010473m	Pru p 2.01	*Prunus persica*	In vitro antifungal activity	Palacin et al., 2010	[49]
			Endo-β-1,3-glucanase activity	Palacin et al., 2010	[49]
Prupe-ppa010522m	Pru p 2.02	*Prunus persica*	In vitro antifungal activity	Palacin et al., 2010	[49]
			Endo-β-1,3-glucanase activity	Palacin et al., 2010	[49]
Prupe-ppa010621m	Pru p 2.03	*Prunus persica*	In vitro antifungal activity	Palacin et al., 2010	[49]
			Endo-β-1,3-glucanase activity	Palacin et al., 2010	[49]
Soldu-10445203	Soldu osm	*Solanum dulcamara*	Antifreeze activity	Newton and Duman, 2000	[89]
Solly-19315	p23	*Solanum lycopersicum*	Transgenic antifungal protection	Fagoaga et al., 2001	[90]
Solly-6093527	NP24	*Solanum lycopersicum*	Endo-β-1,3-glucanase activity	Grenier et al., 1999	[79]
			In vitro antifungal activity	Pressey, 1997	[91]
			Tridimensionnal structure	Ghosh et al., 2008	[17]
Solni-19401631	SnOLP	*Solanum nigrum*	In vitro antifungal activity	Campos et al., 2008	[92]
Thada-121945718	Thaumatin I	*Thaumatococcus daniellii*	Transgenic antifungal protection	Rajam et al., 2007	[16]
			Tridimensionnal structure	Ogata et al., 1992	[93]
Thada-512363	Thaumatin II	*Thaumatococcus daniellii*	Transgenic antifungal protection	Schestibratov and Dolgov, 2005	[94]
Triae-110836639	TLX1	*Triticum aestivum*	Xylanase inhibition	Fierens et al.,2007	[12]
			Tridimensionnal structure	Fierens et al., 2009	[19]
Triae-20257409	Was-3a	*Triticum aestivum*	In vitro antifungal activity	Kuwabara et al., 2002	[95]
Trica-91077308	thaumatin-1	*Tribolium castaneum*	In vitro antifungal activity	Altincicek et al., 2008	[96]
Vitvi-GSVIVT00001103001		*Vitis vinifera*	In vitro antifungal activity	Monteiro et al., 2003	[41]
Vitvi-GSVIVT00001105001		*Vitis vinifera*	In vitro antifungal activity	Monteiro et al., 2003	[41]
Zeama-GRMZM2G374971_T01	Zeamatin	*Zea mays*	Membrane permeabilization	Roberts and Selitrennikoff, 1990	[97]
			ɑ-amylase/trypsin inhibition	Schimoler-O'Rourke et al., 2001	[13]
			In vitro antifungal activity	Huynh et al., 1992	[98]
			Tridimensionnal structure	Batalia et al., 1996	[99]
